# Differential Effects of Power Rehabilitation on Physical Performance and Higher-level Functional Capacity among Community-dwelling Older Adults with a Slight Degree of Frailty

**DOI:** 10.2188/jea.17.61

**Published:** 2007-04-10

**Authors:** Atsuhiko Ota, Nobufumi Yasuda, Shunichi Horikawa, Takashi Fujimura, Hiroshi Ohara

**Affiliations:** 1 Department of Public Health, Kochi Medical School.; 2 Department of Health and Welfare, Kochi City Office.

**Keywords:** effectiveness, older people, Power Rehabilitation, nonrandomized controlled interventional trial

## Abstract

**BACKGROUND:**

Evidence is still insufficient regarding the effects of Power Rehabilitation (PR) on physical performance and higher-level functional capacity of community-dwelling frail elderly people.

**METHODS:**

This nonrandomized controlled interventional trial consisted of 46 community-dwelling elderly individuals with light levels of long-term care needs. They were allocated to the intervention (I-group, n = 24) and control (C-group, n = 22) groups. Of them, 32 persons (17 in the l-group; 15 in the C-group) (median age, 77 years; sex, 28% male) completed the study. The l-group subjects underwent PR twice a week for 12 weeks. The outcomes were physical performance (muscle strength, balance, flexibility, and mobility) and higher-level functional capacity as evaluated by the Tokyo Metropolitan Institute of Gerontology Index of Competence (TMIG-IC) and the level of long-term care need as certified by the public long-term care insurance.

**RESULTS:**

The l-group demonstrated a significant improvement in the measured value of the timed up-and-go test (median change, a decrease of 4.4 seconds versus a decrease of 0.2 seconds, p = 0.033) and the timed 10-meter walk (a decrease of 3.0 seconds versus an increase of 0.2 seconds, p = 0.007) in comparison with the C-group. No significant change was observed in the TMIG-IC scores or in the level of long-term care need in the l-group.

**CONCLUSION:**

PR improved mobility of community-dwelling frail elderly people; however, such improvement did not translate into higher-level functional capacity. Our findings demonstrate the difficulty in transferring the positive effects associated with PR into an improvement in higher-level functional capacity.

An increase in the number of frail elderly people threatens the sustainable management of the public long-term care insurance (PLCI). As a result, there is an urgent need to introduce community health services that are effective in reducing long-term care use by frail elderly.^1^ Although researchers have investigated a variety of physical training methods as the possible preventive measures, questions regarding the effectiveness of them still remain.

Progressive resistance training (PRT), in which the resistance against which muscle generates force progressively increases over time,^2^ has been strongly advocated as a preventive therapy against sarcopenia. Sarcopenia, i.e., an age-related decline in the muscle mass and function,^2^^,^^3^ has attracted attention as a modifiable condition that increases the risk of long-term care use. Dozens of randomized controlled trials (RCTs) to examine the effectiveness of PRT have thus been conducted in developed countries other than Japan. A recent meta-analysis4 based on 62 RCTs for older people with various health statuses concluded that PRT had a positive effect on both muscle strength and gait speed; however, such positive effects on impairment and functional limitation outcomes did not translate into any reduction in physical disability. So far only a few RCTs of exercise training in older Japanese people have been carried out and such studies generally evaluated exercise programs in which resistance training was combined with balance training and aerobic exercises.^5^^,^^6^ Therefore, the effectiveness of resistance training alone is still unclear regarding elderly Japanese individuals.

Recently some researchers have begun to focus attention on power, i.e., the rate of doing work,^7^ to improve physical performance and maintain higher-level functional capacity of frail elderly. To gain power, power training (PT) utilizes a light resistance which is moved at a fast velocity.^8^ Miszko et al^8^ reported in an RCT that PT improved physical performance, especially balance and coordination, endurance, and upper-body flexibility, of community-dwelling older individuals. However, to our knowledge, there so far has been no study describing the effectiveness of PT on maintaining higher-level functional capacity in the elderly.

Meanwhile, Power Rehabilitation (PR),^9^ which employs machine training with light resistance, is a supervised training specifically developed for elderly individuals by Takeuchi and colleagues. Takeuchi^9^ hypotheses that (1) PR induces an advance in power and (2) this change results in an improvement of physical performance and higher-level functional capacity of frail elderly individuals. PR is now used at many care facilities, although these hypotheses have not yet been thoroughly investigated. Previous studies have shown that PR improves physical performance and the level of long-term care need not only in frail elderly inpatients at a geriatric care facility^10^ but also in elderly outpatients.^11^ Because these previous studies possessed no comparison groups, it is actual that evidence only at low levels has accumulated on the effectiveness of PR.

The authors conducted a nonrandomized controlled interventional trial to examine the effect of PR on physical performance (primary outcomes: measurements of impairments and functional limitations according to Nagi's model^12^) and higher-level functional capacity (secondary outcomes: measurements of disability according to Nagi's model^12^) of community-dwelling older people who were judged to require light levels of long-term care under the PLCI.

## METHODS

### Subjects

In the beginning of July 2003, the authorities of Kochi City, Japan, recruited the study participants from the primary insured people under the PLCI who were living in the study district. The district was chosen because the municipal health and welfare center located there was equipped with the training machines utilized for this study. A total of 294 persons were identified as satisfying the following entry criteria: aged 65 years or older; certified for long-term care need at the levels of requiring support, long-term care 1 or 2; taking neither commuting rehabilitation service nor home-visit rehabilitation service; having no subjective symptoms; and their family and doctors both agreeing to let the subject participate in this study. The authors sent each family physician a written outline of PR for their help to medically determine whether their patient was able to perform PR. Totally, 46 gave written consent to participate in this study. They were randomly assigned to the intervention (I-group, n = 24) and control (C-group, n = 22) groups at first. However, we were obliged to change a part of the random allocation afterward. According to the subject's or their family's requests, three subjects allocated to the I-group were replaced with three subjects from the C-group of the same sex, the same level of long-term care need, and a similar age. As a result, the final group allocation was nonrandomized.

Of the 46 participants, 14 (7 in each of the I- and C-groups) dropped out during the intervention period as follows: (1) Seven (four in the I-group and three in the C-group) were absent from the baseline survey for non-health-related personal reasons. (2) Three I-group participants did not complete the intervention; two were hospitalized due to medical conditions that were not related to PR and one withdrew due to a personal reason. And (3) Four participants in the C-group were absent from the follow-up measurement for non-health-related personal reasons. The remaining 17 in the I-group (71% of the original I-group members) and 15 in the C-group (68% of the original C-group members) completed this study and were analyzed as the study subjects.

### Intervention

The I-group subjects executed PR twice a week for 12 weeks (24 sessions) between September and November 2003 in the municipal health and welfare center. The Kochi City authorities transported the I-group subjects with a pickup bus because a walk to and from the study site might work as a training opportunity for them. If the I-group subjects had obtained such an opportunity, then it would have been difficult to evaluate the intervention effect specifically derived from PR. PR training employed Compass^®^ training machines (Proxomed Medizintechnik Inc., Alzenau, Germany) and was performed under supervision by municipal health care professionals, accompanied by warming-up and cooling-down exercises. The weight-machine exercise consisted of horizontal leg press, leg extension/flexion, torso extension/flexion, rowing multifunction, chest press, and hip abduc-tion/adduction.^13^ The subjects spent the first 6 sessions learning the way to use the training machines and adjusting their weight. After performing each machine exercise one set of ten repetitions, the rating of perceived exertion was assessed using the Borg scale.^14^ The weight was adjusted so that the subject felt weak to accomplish the machine exercise. During the remaining 18 sessions, the subjects performed each machine exercise consisting of three sets of ten repetitions. No adverse effect was reported by the participants.

During the intervention period, not only the I-group subjects but the C-group subjects were requested to maintain their usual lifestyle as before the intervention period. They were instructed not to engage in any other exercise programs.

### Measures

At baseline (T1) and immediately after the completion of the 12-week PR training (T2), the subjects were examined regarding their body mass index (BMI), need for assistance in walking, physical performance, and higher-level functional capacity.

Physical performance measurement included muscle strength measures (grip and lower-limb strength), balance measures (timed one-legged standing with open eyes and functional reach^15^), a flexibility measure (sit-and-reach test), and mobility measures (timed up-and-go test^16^ and timed 10-meter walk). Grip strength was assessed with a digital squeeze dynamometer (T.K.K. 5401, Takei Scientific Instruments Co., Ltd, Niigata, Japan). Lower-limb strength was isometric knee extensor strength with the knee flexed to 90 degrees. Its measurement was conducted with a hand-held dynamometer (a manual muscle testing sensor, EG-230, SAKAI Medical. Co., Ltd, Tokyo, Japan). For these two measurements, the mean of measured values of both sides was entered into the analysis. The functional reach was gauged with a reach meter (CK-101, SAKAI Medical. Co., Ltd, Tokyo, Japan). For the timed up-and-go test and timed 10-meter walk, use of assist in walk was permitted according to the subject's demand. The physical performance tests were not executed in case where possible accidents could occur due to the subject's physical status. To minimize the systematic and random variations of measured values that might distort the group comparisons, the same health care professional was assigned to measure at both T1 and T2. The authors trained the examiner how to conduct the physical performance measurements.

Higher-level functional capacity was evaluated based on the Tokyo Metropolitan Institute of Gerontology Index of Competence (TMIG-IC)^17^ and the level of long-term care need certified by the PLCI. The TMIG-IC consists of the following three subscales: instrumental self-maintenance (5 items), intellectual activity (4 items), and social role (4 items). For the total and each subscale score, a greater score indicates a higher capacity. To determine the level of long-term care need, the PLCI regulates the computerized judgment based on the standardized interview survey on the insured's physical and mental conditions.^1^ The registered nurses who were working for the Kochi City authorities and possessed much practical experience were assigned to carry out the survey.

### Ethics

The study protocol was approved by the Ethical Committee of Kochi Medical School. After this study period, the Kochi City authorities offered the C-group subjects the free opportunity to undergo PR training.

### Statistical Analysis

Differences in the baseline characteristics was examined with the Mann-Whitney U test for continuous variables and categorical variables with orders and with the Fisher exact test for 2×2 categorical variables between the 17 I-group subjects who completed the 12-week intervention and the 15 C-group subjects who attended both T1 and T2 measurements. The same analysis was also performed between the 32 study subjects and the 14 drop-outs to compare their age, sex, and long-term care level.

Regarding the change in the value of each measurement between T1 and T2, statistical significance was assessed with the Mann-Whitney U test between the I- and C-groups. The Fisher exact test was used to compare the proportion of those with an improvement in the measured value during the intervention period between the two groups. In this analysis, the subjects with missing value at T1 and/or T2 were regarded as experiencing no improvement in the variable. This analysis was also performed, assuming that all the drop-outs were regarded as experiencing no improvement. Multiple logistic regression analysis was fit to control age, sex, long-term care level at T1, and assist in walk at T1 in comparing the odds of improvement between the 17 I-group subjects and 15 C-group subjects.

The level of significance was 0.05 (two-tailed) for all tests. SPSS® 13.0J for Windows (SPSS Japan Inc., Tokyo, Japan) was used for the statistical analysis.

## RESULTS

### Baseline Variables

The median age of the 32 study subjects who completed the study was 77 years old and the proportion of male subjects was 28 %.

Between the I- and C-groups, there was no significant difference in baseline characteristics with the exception of measured value of the timed 10-meter walk (Table 1). It was slower in the I-group than in the C-group, although the difference showed only borderline significance. Although all subjects were certified users of long-term care at the time of recruitment, three I-group subjects and two C-group subjects were not eligible for the certification of long-term care use at T1.

**Table 1.** The subjects' characteristics at baseline (T1).

Variables [frequency (%) or median (range)]	Intervention group (n=17)	Control group (n=15)	P
Age (year)	77 (67, 98)	82 (65, 87)	0.733
75 or older	10 (59%)	10 (67%)	0.726

Sex: Male	5 (29%)	4 (27%)	1.000

Body Mass Index (kg/m^2^)	22.9 (19.8, 30.6)	22.0 (16.6, 30.5)	0.485
25 or more	7 (41%)	4 (27%)	0.472

Use of assistance in walking	12 (71%)	7 (47%)	0.153

Physical performance			
Grip strength (kg)	14.3 (6.3, 26.3)	14.7 (9.8, 27.7)	0.763
Lower-limb strength (kgf)	14.9 (6.5, 22.6)	17.2 (5.3, 28.1)	0.737
One-legged standing (sec)	4.5 (0.9, 5.9)^*^	3.8 (1.0, 13.9)^†^	0.734
Functional reach (cm)	22.5 (8.5, 32.5)	16.0 (7.5, 30.5)	0.241
Sit-and-reach test (cm)	16.0 (5.5, 38.5)	16.5 (2.0, 36.5)	0.508
Up-and-go test (sec)	22.1 (11.6, 52.4)	20.0 (11.5, 71.2)	0.317
Timed 10m walk (sec)	14.4 (7.7, 37.0)	10.5 (8.2, 32.8)	0.062

Score of TMIG-IC			
Total	8.0 (4, 12)^*^	9.0 (2, 12)	0.643
Instrumental self-maintenance	4.0 (1, 5)^*^	4.0 (0, 5)	0.380
Intellectual activity	3.0 (1, 4)^*^	3.0 (1, 4)	0.623
Social role	2.5 (0, 3)^*^	2.0 (0, 4)	0.874

Long-term care level			
Non applicable	3 (18%)	2 (13%)	0.755
Support-required	8 (47%)	9 (60%)	
Care level 1	5 (29%)	4 (27%)	
Care level 2	1 (6%)	0 (0%)	

Note. TMIG-IC: the Tokyo Metropolitan Institute of Gerontology Index of Competence. P value was calculated with the Mann-Whitney U test for continuous variables and categorical variables with orders and with the Fisher exact test for 2 x 2 categorical variables.

*, **†** : n = 14 and 13, respectively, due to a missing response.

Between the 32 study subjects and the 14 drop-outs, the proportion of those aged 75 or older was higher among the drop-outs (93%) than among the subjects (63%), and this difference was marginally significant (p = 0.072). There was no significant difference in the distribution of sex and long-term care level.

### Change in Physical Performance (Tables 2 and 3)

**Table 2.** Measured value of physical performance at T2 and its change between T1 and T2 [median (range)]: comparison between the intervention (n = 17) and control (n = 15) groups.

Physical performance	Measured value at T2		Change		
	
	Intervention	Control	Intervention	Control	P
Grip strength (kg)	16.5 (7.1, 31.8)	14.9 (11.1, 32.2)	1.7 (-0.8, 12.6)	1.1 (-1.7, 4.5)	0.186
Lower-limb strength (kgf)	22.0 (11.8, 36.7)	20.4 (10.2, 26.2)^*^	3.8 (-0.7, 22.9)	4.5 (-1.9, 8.9)^*^	0.275
One-legged standing (sec)	2.9 (0.7, 30.4)^†^	3.5 (0.7, 18.9)^†^	-0.7 (-11.3, 16.9)^*^	-0.2 (-3.4, 15.3)^‡^	0.560
Functional reach (cm)	22.0 (9.5, 33.5)	16.0 (6.0, 30.0)	0.5 (-5.5, 9.0)	-1.5 (-7.5, 7.5)	0.036
Sit-and-reach test (cm)	20.0 (7.0, 39.0)	16.5 (3.5, 34.0)^*^	2.5 (-8.0, 20.0)	0.5 (-3.5, 5.5)^*^	0.061
Up-and-go test (sec)	16.4 (8.4, 52.8)	17.1 (12.4, 66.0)	-4.4 (-14.4, 24.2)	-0.2 (-9.1, 4.3)	0.033
Timed 10m walk (sec)	11.4 (6.1, 36.7)	10.4 (6.4, 42.8)	-3.0 (-10.0, 8.0)	0.2 (-8.6, 10.0)	0.007

Note. Change = (measured value at T2) - (measured value at T1). P value was calculated with the Mann-Whitney U test.

*, † , ‡ : n = 14, 16, and 13, respectively, due to a missing execution.

**Table 3.** The number (%) of the subjects with improvement in measured value of physical performance between T1 and T2: comparison between the intervention (n = 17) and control (n = 15) groups.

Physical performance	Intervention	Control	Rate ratio (95% CI)	P
Grip strength (kg)	16 (94)	13 (87)	1.09 (0.86-1.37)	0.589
Lower-limb strength (kgf)	16 (94)	11 (73)	1.28 (0.92-1.78)	0.133
One-legged standing (sec)	4 (24)	5 (33)	0.71 (0.23-2.16)	0.699
Functional reach (cm)	9 (53)	3 (20)	2.65 (0.88-8.01)	0.076
Sit-and-reach test (cm)	13 (76)	9 (60)	1.27 (0.78-2.08)	0.450
Up-and-go test (sec)	16 (94)	8 (53)	1.76 (1.08-2.88)	0.013
Timed 10m walk (sec)	16 (94)	7 (47)	2.02 (1.16-3.51)	0.005

Note. Subjects with missing data were regarded as experiencing no improvement. P value was calculated with the Fisher exact test.

CI: confidence interval

There was a significant difference between the I- and C-groups in the change in the measured values of the timed up-and-go test and the timed 10-meter walk between T1 and T2. The ratio of proportions of those with an improvement in the measured values was significantly different. The significance remained even after controlling age, sex, long-term care level at T1, and assist in walk at T1 (p = 0.030 and 0.032, respectively). When all the drop-outs were included into the analysis, a marginally significant difference in the proportions of those with the improved measured value was found for the timed up-and-go test (p = 0.075) and a significant one was observed for the timed 10-meter walk (p = 0.038).

Regarding the functional reach, there was a significant difference between the two groups in the change in the measured values between T1 and T2. The difference in the proportion of those with the improved measured value between the two groups was marginally significant. The difference turned significant when controlling age, sex, long-term care level at T1, and assist in walk at T1 (p = 0.040). The same result was also found when entering the drop-outs into the analysis (p = 0.096).

A borderline significant difference was seen in the change in the measured values of the sit-and-reach test during the intervention period between the two groups. However, there was no significant difference in the proportion of those with the improved measured value.

For grip, lower-limb strength and timed one-legged standing, there was no significant difference between the two groups in the change in the measured values during the intervention period and in the proportion of those with the improved measured values.

### Change in Higher-level Functional Capacity

There was no significant difference between the two groups in the change in either the total TMIG-IC score or each of the subscale scores between T1 and T2 (Table 4). There was no significant difference in the proportion of those with an improvement in the total and each subscale scores between the I- and C-groups (Table 5). This result was unchanged as the drop-outs were included into the analysis.

**Table 4.** Scores of the Tokyo Metropolitan Institute of Gerontology Index of Competence (TMIG-IC) at T2 and its change between T1 and T2 [median (range)]: comparison between the intervention (n = 17) and control (n = 15) groups.

Score of TMIG-IC	Measured value at T2		Change		
	
	Intervention	Control	Intervention	Control	P
Total	10.0 (4, 12)^*^	10.0 (3, 13)	0 (-1, 6)^†^	1.0 (-1, 5)	0.339
Instrumental self-maintenance	4.0 (2, 5)^*^	4.0 (0, 5)	0(0, 3)^†^	0 (-1, 2)	0.346
Intellectual activity	3.0 (1, 4)^*^	4.0 (1, 4)	0 (-2, 1)^†^	0 (-2, 2)	0.357
Social role	2.0 (0, 4)^*^	2.0 (0, 4)	0 (-2, 3)^†^	1.0 (-2, 2)	0.143

Note. Change = (measured value at T2) - (measured value at T1). P value was calculated with the Mann-Whitney U test.

*, † : n = 14 and 13, respectively, due to a missing response.

**Table 5.** The number (%) of the subjects with improvement in the scores of the Tokyo Metropolitan Institute of Gerontology Index of Competence (TMIG-IC) and in the level of long-term care need between T1 and T2: comparison between the intervention (n = 17) and control (n = 15) groups.

	Intervention	Control	Rate ratio (95% CI)	P
Score of TMIG-IC				
Total	4 (24)	8 (53)	0.44 (0.17-1.17)	0.144
Instrumental self-maintenance	4 (24)	4 (27)	0.88 (0.27-2.93)	1.000
Intellectual activity	2 (12)	5 (33)	0.35 (0.08-1.56)	0.209
Social role	3 (18)	8 (53)	0.33 (0.11-1.03)	0.061

Level of long-term care need	4 (24)	4 (27)	0.88 (0.27-2.93)	1.000

Note. Subjects with missing data were regarded as experiencing no improvement. P value was calculated with the Fisher exact test.

CI: confidence interval

No significant difference was observed in the proportion of those with an improvement in the level of long-term care need during the intervention period between the two groups (Table 5). Entering the drop-outs into the analysis did not change this finding.

## DISCUSSION

Regarding the findings in this nonrandomized controlled interventional study, PR improved the mobility measurements (timed up-and-go test and timed 10-meter walk). Moreover, PR was suggested to ameliorate a balance measurement (functional reach). Previous uncontrolled trials of PR reported a positive effect on physical performance, which was confirmed by the present findings, especially on mobility. According to a meta-analysis by Latham et al,^4^ no positive effect of PRT was observed on the timed up-and-go test. The change in both domains of walking speed and dynamic balance might thus contribute to an improvement in the timed up-and-go test composed of posture alteration (chair-rising/sitting and turn-around) and walking.

No significant change was observed on muscle strength and flexibility. In the present study, the load of PR might be inadequate to yield a greater gain of muscle strength than the extent of natural variation of muscle strength that occurred during the intervention period. The characteristics of the subjects also might account for the null effect on strength. Most subjects were certified users of long-term care services and were functionally limited. According to a subgroup analysis in a meta-analysis on PRT,^4^ the effects on muscle strength were small in older people with physical disabilities or functional limitations.

Previous uncontrolled trials of PR showed positive effect on both physical performance and higher-level functional capacity. However, in the present nonrandomized controlled interventional trial, the positive effects of PR on physical performance did not translate into the measurements of higher-level functional capacity, i.e., TMIG-IC score and the level of long-term care need. Several explanations can be offered to account for this pattern of effect. First, the duration of training period might be too short to obtain the desired gains in physical disability outcomes. An intervention period of a longer duration may thus be required to achieve a substantial improvements.^18^ Second, the instruments used to assess disability, especially the TMIG-IC, could not detect any important changes in the disability outcomes either because of ceiling effects or due to the global increments of the item ratings.^18^ Finally, the lack of any effect on the physical disability indicates that improvements in physical performance alone are insufficient to obtain gains in physical disability outcomes. For exercise programs to be effective regarding the physical disability, intervention programs should also address behavioral and social factors. Motivation, self-efficacy, and physical and social environments all need to be considered.^4^^,^^18^ It is interesting to note that the paucity of effect transfer has been reported not only for PRT but for other forms of physical training methods including aerobic exercises and balance training.^18^ Health care professionals who engage in PR therefore need to recognize the difficulty in transferring the positive effects of exercise to the disability outcomes among frail elderly.

The drop-outs included more participants aged 75 or older than those who completed the study. Although the training in the intervention program was supervised by health care professionals, it was difficult to have old-old participants adhere to the exercise program. It would be necessary to clarify in the future what physical or mental conditions of the community-dwelling frail older people obstruct completion of PR training.

This study has several limitations. First, the group allocation was distorted by the exchange of subjects between the I- and C-groups after the random assignment. Although there was no significant difference in the baseline characteristics between the two groups, with the exception of the timed 10-meter walk, the distortion in the group allocation might thus have reduced the comparability of the two groups. Second, the assessor of physical performance, who was not blinded to the group assignment, may have unintentionally overestimated the I-group measurements at T2 while also underestimating the C-group measurements. Third, because the number of the subjects was small, the limited statistical power might have inhibited the detection of small effects of PR, especially on the secondary outcomes. Although the authors found the same pattern of results when regarding all the drop-outs as experiencing no improvement, this assumption could lead an excessive underestimation of the effect of PR training.^19^ Fourth, because the C-group was not an attention control group, the difference in the changes between the two groups was attributable not only to the specific effect of PR but to the effect associated with social interaction with research staff. To differentiate the two effects, the present trial should have possessed an attention control group. Finally, as suggested from the change in grip and lower-limb strength in the C-group during the intervention period, some C-group subjects could have made behavioral alteration that resulted in the improvement in physical performance and higher-level functional capacity. This change could have biased the present findings toward observing no effect. The authors should have individually investigated changes of the subjects in the physical activity and in the ways of living during the study period.

The effect of PR was evaluated in this study by examining the change in physical performance and higher-level functional capacity during a 12-week intervention period. However, the ultimate purpose of such interventional trials is to reduce the risk of deterioration in higher-level functional capacity and postpone its onset. This goal could not be reached for in this study and should be investigated in the future by longitudinal studies which have a longer observation period.
